# Integrating Health Research into Disaster Response: The New NIH Disaster Research Response Program

**DOI:** 10.3390/ijerph13070676

**Published:** 2016-07-04

**Authors:** Aubrey Miller, Kevin Yeskey, Stavros Garantziotis, Stacey Arnesen, April Bennett, Liam O’Fallon, Claudia Thompson, Les Reinlib, Scott Masten, James Remington, Cindy Love, Steve Ramsey, Richard Rosselli, Betsy Galluzzo, Joy Lee, Richard Kwok, Joseph Hughes

**Affiliations:** 1 National Institute of Environmental Health Sciences, Bethesda, MD 20892, USA; 2 MDB, Inc., Washington, DC 20036, USA; kyeskey@michaeldbaker.com (K.Y.); beagin@michaeldbaker.com (B.G.); jlee@michaeldbaker.com (J.L.); 3 National Institute of Environmental Health Sciences, Research Triangle Park, NC 27709, USA; garantziotis@niehs.nih.gov (S.G.); ofallon@niehs.nih.gov (L.O.); thompso1@niehs.nih.gov (C.T.); reinlib@niehs.nih.gov (L.R.); masten@niehs.nih.gov (S.M.); remingtonj@niehs.nih.gov (J.R.); Richard.Kwok@nih.gov (R.K.); hughes3@niehs.nih.gov (J.H.); 4 National Library of Medicine, Bethesda, MD 20892, USA; arneses@mail.nlm.nih.gov (S.A.); lovec@mail.nlm.nih.gov (C.L.); 5 Contractor, National Institute of Environmental Health Sciences, Bethesda, MD 20892, USA; april.bennett@nih.gov; 6 Social & Scientific Systems, Inc., Durham, NC 27703, USA; SRamsey@s-3.com (S.R.); RRosselli@s-3.com (R.R.)

**Keywords:** disasters, disaster research, disaster epidemiology, environmental health, science preparedness, disaster risk reduction, strategic science

## Abstract

The need for high quality and timely disaster research has been a topic of great discussion over the past several years. Recent high profile incidents have exposed gaps in knowledge about the health impacts of disasters or the benefits of specific interventions—such was the case with the 2010 Gulf Oil Spill and recent events associated with lead-contaminated drinking water in Flint, Michigan, and the evolving health crisis related to Zika virus disease. Our inability to perform timely research to inform the community about health and safety risks or address specific concerns further heightens anxiety and distrust. Since nearly all disasters, whether natural or man-made, have an environmental health component, it is critical that specialized research tools and trained researchers be readily available to evaluate complex exposures and health effects, especially for vulnerable sub-populations such as the elderly, children, pregnant women, and those with socioeconomic and environmental disparities. In response, the National Institute of Environmental Health Science has initiated a Disaster Research Response Program to create new tools, protocols, networks of researchers, training exercises, and outreach involving diverse groups of stakeholders to help overcome the challenges of disaster research and to improve our ability to collect vital information to reduce the adverse health impacts and improve future preparedness.

## 1. Introduction

The need for high quality and timely disaster research has been a topic of great discussion over the past several years [1,2,3]. During federally declared disasters, the National Institute of Environmental Health Sciences (NIEHS) is tasked to assist in the federal response efforts, usually by providing either health and safety training or other educational materials for disaster workers. Recent high profile incidents have exposed gaps in knowledge about the health impacts of disasters or the benefits of specific interventions—such was the case with the January 2014 release of a mixture of industrial chemicals into the Elk River affecting the municipal water supply of 300,000 people living in and around Charleston, West Virginia [4]. In this case, as with many others, there was very little information about the toxicity or health impacts of the chemicals involved. The inability to perform timely research to inform the community about health and safety risks further heightened community anxiety and distrust. Recent events associated with lead-contaminated drinking water in Flint, Michigan and the evolving health crisis associated with the emergence of the Zika virus disease underscore the ongoing need for specialized research in disaster settings and the importance of ensuring high-quality and timely data collection. Since nearly all disasters, whether natural or man-made, have an environmental health component, it is critical that specialized research tools be utilized to best understand the health effects that may be seen with disaster-related exposures, especially for vulnerable sub-populations such as elderly, children, pregnant women, and those with socio-economic and environmental disparities. Such observations have galvanized NIEHS to re-double its efforts to improve methods for determination of the health impacts of disasters in order to better monitor and respond to the needs of affected communities.

Much of the existing body of knowledge concerning health effects following disasters has been generated by epidemiologists. In 1976, Lechat articulated what has become the seminal article on disaster epidemiology and generated a call to action for increased study of the health impact of disasters [5]. Subsequent efforts to develop and apply epidemiologic principles to disaster settings have continued to evolve, and were recently summarized by a subcommittee of the Council of State and Territorial Epidemiologists (CSTE) [6]. The subcommittee developed a framework for applying epidemiologic principles to public health preparedness and focused on four methodologic areas: survey techniques, public health surveillance and tracking, epidemiological investigations, and longitudinal investigations. These methods, along with the efforts of chemists and toxicologists, comprise the foundation for environmental health research, defined as scientific inquiry designed to better understand the acute and long-term health effects associated with environmental exposures.

Short-term benefits of disaster research include accurately characterizing the scope and nature of exposures of concern; understanding acute health impacts; identifying vulnerable populations; improving worker safety; and determining the effectiveness of interventions. Long-term benefits include improved understanding of the delayed and chronic health effects from exposures and other disaster related risk factors; increased community resilience; expanded knowledge of the effectiveness of medical countermeasures and other mitigation strategies intended to bolster community health and welfare; and improved health system preparedness, response, and recovery for future events.

While health research has been successfully performed in response to a variety of disasters including the World Trade Center attack, Hurricane Katrina, and the *Deepwater Horizon* Oil Spill, there are a number of difficulties in performing environmental health research in response to disasters [1,7]. Much of the research conducted is convenience-based sampling using non-systematic collection of health information. For reasons to be described later, data collection frequently takes place many months after the event and baseline information is often missing and longitudinal health data are unavailable. Exposure data are often not measured, thereby limiting the ability to detect potential associations between reported health effects and specific exposures. Community input into research topics and protocol design is not typically solicited by researchers and as a result may not address the health issues important to those most affected [7]. Such difficulties weaken the study design and any conclusions to be drawn concerning the health effects and risks from exposures. Thus, while the infrastructure frequently exists to support health surveillance and acute epidemiological investigations (e.g., cross-sectional surveys), the resources, processes, and information necessary for actually conducting timely disaster research remain largely inaccessible to public health responders and the academic community.

## 2. Challenges to Disaster Research

Despite the growing interest in understanding the health impacts of disasters, critical data and opportunities to answer important questions of concern continue to be lost due to persistent delays in implementing timely field investigations.

**Figure 1:**
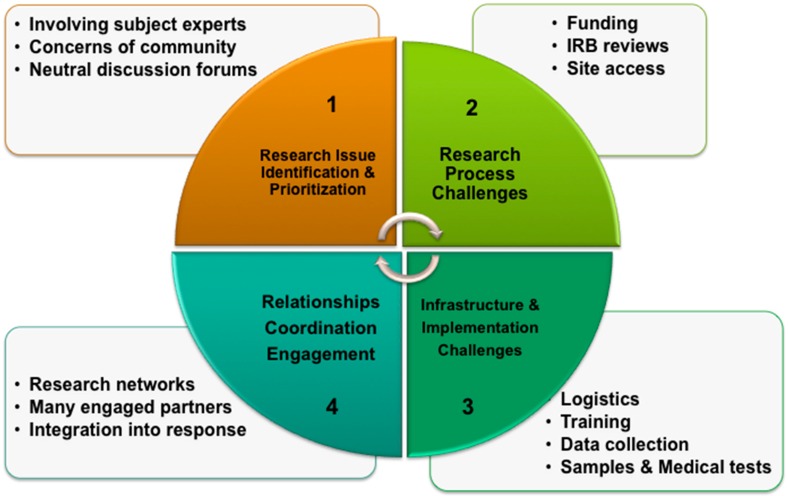
Major categories of challenges for conducting disaster research.

Key challenges to conducting early disaster research fall into four major categories (Figure 1).

### 2.1. Research Issue Identification and Prioritization

The ability to rapidly identify and prioritize data gaps and post-disaster research questions continues to be a challenge for health officials and others involved in managing the situation and recovery efforts. Often, when a disaster occurs, multiple agencies and experts independently begin to evaluate the situation, including issues of concern, available information, and knowledge gaps. Research efforts are subject to being fragmented, uncoordinated, or ineffective due to the inability to prioritize research resources.

Although a secondary issue, until recently, there has been no organized infrastructure or forums for interested parties to coordinate and share information that can lead to conflicting messages and risk communication from health officials to the community—such was the case with the Ebola Virus Disease response, where data gaps concerning the risks for Ebola transmission, and effective venues for sharing of knowledge and information, contributed to ambiguous recommendations for health care workers [8]. Additionally, the absence of coordination can lead to conflicting messages that impede accurate risk communication from health officials to the community.

### 2.2. Research Process Challenges

The ability to create timely “processes to support research” such as rapid funding, expedited Institutional Review Board (IRB) reviews of research protocols, and authorized access to impacted sites and populations remains a challenge.

Following a disaster, there are significant impediments to obtaining and distributing needed funding to disaster researchers. The usual federal funding source for response efforts, the Stafford Act, is legally bound to address activities directly related to the acute response and has never been used to fund health-related research activities [9]. Supplemental federal appropriations following Hurricane Sandy took over 60 days for congressional approval. Federal organizations typically require weeks to process supplemental appropriations before release, even under expedited conditions. When funding becomes available, the competitive grants process is not supportive of rapid research. For example, the well-intentioned NIEHS Time-Sensitive grants program (R-21) may require up to twelve weeks from application to award [10]. Following the appropriation of federal funds after Hurricane Sandy, the U.S. Department of Health and Human Services (HHS) used a competitive process to award funds for research. However, that initial process took nearly one year following the event—a timeline that is acceptable for retrospective studies but is unsuitable for early collection of time-sensitive data and prospective studies. While the HHS efforts are an important step forward in disaster research, they illustrate the bureaucratic delays in traditional funding of disaster research. Efforts need to continue to develop timely funding mechanisms for federal health agencies to provide support for extramural research, similar to the National Science Foundation’s (NSF) Grants for Rapid Response Research (RAPID) that enables program managers to initiate funding within days of an event [11].

Another cause for delay is the research protocol review process. Ensuring adequate human subject protections and provisions for obtaining informed consent are fundamental steps in the human research protocol approval process. It is also essential that research protocols have a sound scientific rationale, which requires a comprehensive scientific review. Traditionally, review begins as a study is proposed and no research takes place until the protocol is determined to be scientifically sound and protective of any human or animal subjects. IRBs have historically been reluctant to pre-approve protocols and even expedited reviews can take weeks for IRB approval, which prevents timely collection of important perishable post-disaster data. As most disasters are unexpected, the only reliable way to conduct timely research is to develop processes for incorporating reviewing bodies into disaster research efforts prior to the occurrence of an event.

Access to the immediate disaster environment poses another substantive challenge for timely research. The post-disaster environment may be so hazardous that research personnel may not be permitted access to it for safety reasons for several weeks. Emergency management officials who are appropriately focused on immediate life-saving and emergency response activities may believe that researchers could interfere with those activities and that they would require assistance, consume limited resources, and over-burden the damaged infrastructure. Similarly, public health officials who are engaged in response effort may not have the resources, or recognize the value of supporting research activities while life-saving needs of the affected population are at stake. For disaster researchers to obtain rapid access to the disaster site and exposed populations, incident managers will need to better understand the ability and importance of the research team in supporting response and recovery activities, answering questions from the community, building community trust among the responders and researchers, creating enhanced community resilience, and better preparing for the next event. To be least burdensome and most successful, the research efforts for an event need to be considered as early as possible, integrated into the response with senior officials, and effectively engage all the interested parties in the development and implementation of the studies.

### 2.3. Research Infrastructure and Implementation Challenges

The lack of available data collection tools, supporting documentation, and standing infrastructure to support the field implementation of research contributes to delays in timely data collection following a disaster. Such needs include the availability of research protocols with pre-reviews or approvals from an IRB, data collection instruments, consent forms, medical testing and exposure sampling equipment, instructional manuals, data management systems, and biospecimen collection supplies.

Research efforts are further delayed by the lack of research staff with training in two key areas: health and safety, and incident management. Ideally, researchers would have some training completed in advance and receive just-in-time training that is mission specific prior to deployment. Advance training can serve to mitigate some of the risks associated with a dangerous and austere disaster site and can assist researchers in gaining a better understanding of the response structure used by first responders. The health and safety of disaster workers must take precedence over all other missions. General worker safety and health training can be provided at the beginning of any deployment, but specialized training such as Hazardous Waste Operations and Emergency Response (HAZWOPER) can take up to 40 h. Training researchers to function within the response infrastructure will build trust and credibility with other traditional response partners. Training on the research protocol, data collection methods, and basic mission orientation can take additional time, which can delay prompt deployment of research resources and result in lost opportunities to collect perishable information. Having training materials prepared prior to the disaster, requiring minimal customization to tailor them to the event, will enable just-in-time training of research responders and a faster response time.

### 2.4. Engagement and Coordination with Stakeholders and Integration into Response Plans

The effective engagement of key stakeholders, including those impacted by an incident is essential to support the implementation of research. Additionally, engagement must include discussions on how researchers and research activities can be integrated into the acute response plans both locally and at the federal level.

Effective responses are characterized by trusting relationships of responders working collaboratively to develop comprehensive response and recovery plans. The best way to build this professional trust is by working together before an incident occurs and the same is true for planning for disaster research. Disaster research requires the collaboration of professionals from public health, emergency management, academia, and private industry, i.e., the “whole of community” approach espoused by the Federal Emergency Management Agency (FEMA). However, this collaboration requires the input of citizens in developing a research portfolio based on community vulnerabilities and hazards and addresses the needs of the entire community.

## 3. NIH Disaster Research Response Program: An Environmental Health Research Initiative

In September 2013, the National Institutes of Health (NIH) initiated a program that serves as a model for disaster research programs. The NIH Disaster Research Response Program (DR2) began as a pilot project sponsored by the National Institute of Environmental Health Sciences (NIEHS) and the National Library of Medicine (NLM). Based on the gaps in, and challenges to, the conduct of disaster research described above, DR2 focuses on six key objectives to enable more timely disaster research while also facilitating a broader discussion to advance our national disaster research capabilities. The key objectives include: (1) identification of important research questions and priorities; (2) improved access to data collection tools for researchers; (3) improved NIEHS capability to quickly collect data; (4) trained researchers versed in disaster tools; (5) integration into preparedness, response, and recovery systems; and (6) research processes that include public health, academia, and impacted workers and communities.

**(1)**  **Rapid identification of important environmental health research questions and priorities**

In response to these challenges, new models for identifying data gaps and research priorities have been started. DR2 has supported workshops hosted by the National Academies of Sciences, Engineering, and Medicine to discuss health issues related to the *Deepwater Horizon* Oil Spill and the current Zika outbreak [12,13]. As part of the Hurricane Sandy Response, NIEHS participated in the U.S. Department of the Interior’s Strategic Sciences Group (SSG), which provides a standing capacity to rapidly assemble teams of scientists to construct interdisciplinary scenarios including the data needs and science concerns for environmental crises [14]. Beyond identifying research priorities, there is the need to simultaneously assess various factors (e.g., available resources, statistical power, health significance, generalizability, available exposure data) to determine valid and useful scientific studies that can be conducted to answer the research hypotheses of interest. To help further such considerations, the National Institute of Occupational Safety and Health (NIOSH) has developed a framework to help determine when to conduct responder health research following disasters [15].

**(2)**  **Timely access to data collection tools for researchers**

One of the key principles of DR2 is to empower state and local responders to conduct and sustain research efforts within their jurisdiction, especially in those disasters that do not require federal involvement. This approach is exemplified by the creation of a publicly accessible repository of data collection tools and research protocols, as well as other information to support disaster research, hosted by the NLM on an evolving National Institutes of Health (NIH) DR2 website that was initiated in January 2015 [16]. This information is intended to help researchers from any community to rapidly obtain various data collection tools that have been used previously in research efforts. Researchers can use or modify these instruments in accordance with their needs.

The development of the DR2 repository was initiated by a literature search of over 10,000 peer-reviewed articles and numerous websites to identify questionnaires, surveys, and other instruments used to support disaster research. The review process revealed tools that were acceptable for inclusion into the DR2 repository. Specific metadata were then generated for each of the data collection tools, using various descriptive fields for the information, to help standardize and improve the selection of an appropriate tool by the research community (Table 1). Additionally, filters were created to allow the refinement of search results to facilitate easy identification of these tools. Currently, the NIH DR2 website hosts data collection instruments used to measure post-disaster impacts in eight research categories including: environmental exposures, lifestyle and quality of life, mental health and cognitive function, occupational health, preparedness, social support and resiliency, specific body systems, and disaster-specific health concerns. Since the disaster research landscape is ever changing, new tools (or links to copyrighted tools) are periodically being added to the repository as they become available. To date, the DR2 website hosts over 200 tools, and we will be adding over a hundred new items for the research community in the coming year.

**(3)**  **Rapid acquisition of pre-and post-incident disaster data**

A scalable research protocol, called “Rapid Acquisition of Pre-and Post-Incident Disaster Data” (RAPIDD), was developed by NIEHS to facilitate the collection of epidemiologic information, laboratory test results, and the collection and storage of human bio-specimens. RAPIDD was based, in-part, on the NIEHS GuLF STUDY protocol used for environmental health research of over 32,000 disaster clean-up workers who responded to the 2010 *Deepwater Horizon* Oil Spill [17]. Prior to release, the RAPIDD protocol underwent peer and scientific review and was submitted to the NIEHS IRB for approval. The peer-review process consisted of discussions, and one training exercise, with various stakeholders including academic researchers, public health, emergency management, and first responders. After reviewing the protocol, exercise participants were asked to comment on incentives and barriers for participating in the protocol, the informed consent process, and the ease of use of the protocol. All feedback was incorporated into the protocol, and it was submitted to the NIEHS IRB, which provisionally approved the RAPIDD protocol in May 2015 for use in post-disaster settings [18]. Final approval will be based on an expedited amendment that includes clarification of the disaster type, target sample size, exposures and potential health effects of interest, specific questionnaires, and procedures that will be implemented.

Additionally, the DR2 team is providing technical assistance to extramural researchers interested in developing their own protocols or implementing a research response to local disasters. For instance, during recent flooding, oil and gas releases, and factory fires, DR2 team members helped academic and public health researchers with access to relevant questionnaires and tools, provided guidance on using the RAPIDD protocol, and assisted with IRB-related matters. The DR2 team now tracks all requests for assistance, support provided, and final disposition of the request to help guide further improvements.

In collaboration with DR2, the NIEHS National Toxicology Program (NTP) has been making new inroads to applying various toxicology tools and assets to improve its capacity and capability, from rapidly assessing the available toxicology literature, to coordinating the identification of toxicology research issues and gaps, as was done in response to the *Deepwater Horizon* Oil Spill and the Elk River chemical spill. In the latter example, the NTP conducted a number of short-term studies to provide information relevant to exposures of local residents to 4-methylcyclohexanemethanol for government officials and decision-makers [19].

**(4)**  **Trained researchers versed in disaster tools and issues that can perform or support science investigations**

Training a network of environmental health researchers that can effectively and safely contribute to post-disaster research efforts has been a cross-cutting imperative of the DR2 program. Central to this effort has been the execution of tabletop exercises in two locations, Los Angeles, CA and Houston, TX, that used scenarios based on local hazards and vulnerabilities. Local public health and academic organizations were asked to participate in the planning process. Prior to each exercise, DR2 team members met with local, state, and federal response officials and academic faculty to develop exercise objectives, conduct site visits to the “affected” areas, and discussed key issues in the exercise scenario. There was an average of 90 exercise participants per exercise representing state, local, and federal partners from, public health, emergency management, public safety, academic centers, private industry, and community activists. Each exercise underwent an evaluation of its effectiveness in raising awareness of the need for disaster research. The exercises demonstrated the need for all stakeholders to collaborate before an event, as many of the potential disaster research responders had not met prior to the exercises. DR2 exercises also highlighted the importance of research efforts supporting public health practice and disaster response and recovery efforts.

Health and safety considerations regarding the deployment of researchers to impacted areas also need to be addressed and included in any protocols and response efforts. The DR2 Program has worked to address this issue by making available relevant information on the NIH DR2 website [16] along with links to situation specific NLM resources, as well as actual on-site training materials and guidance through the NIEHS Worker Training Program [20] and its national clearing house of educational and training information [21]. The mental health impact on responders must also be considered in the health and safety considerations. The Worker Training Program has developed a behavioral health resiliency curriculum that is available to responders who can assist in preparation for deployment. Mental health considerations are an essential component to any deployment curriculum.

**(5)**  **Research response plans and processes that include public health, academia, impacted workers, community groups, and other relevant stakeholders**

Communities may be wary of research when a disaster occurs, so ensuring that researchers work to assist public health in their various missions is crucial for gaining credibility and acceptance from the affected populations. Public health officials and researchers need to clarify the public’s understanding of the importance of research in assisting emergency responders, medical personnel, and public health departments in their various roles. Abara and colleagues discussed how engaging community members in the recovery process following a chlorine spill assisted with focusing state and local public health priorities and ultimately resulted in community-driven public health research [22]. This incident demonstrated that including the community in planning for health consequences of disasters enhanced the full spectrum of response and recovery activities [23]. More importantly, community input helps prioritize research activities and provides the opportunity for the research to address community concerns [24]. Local community organizations have a significant role in determining the resilience to a disaster and are essential to developing preparedness plans, response activities, and recovery priorities. As the DR2 exercises revealed, community advocacy groups are often not consulted or invited to participate in preparedness and response activities, although they may represent the most vulnerable populations.

To help ensure that the affected community, as well as other stakeholders, are included in the DR2 efforts, NIEHS has capitalized on its intramural scientists and extramural academic research centers to establish the Environmental Health Network (EHN). The EHN is intended to help collectively further the goals of the DR2 program through constructive input on the disaster research agenda, repository development, protocol reviews, training materials, and research operations.

**Figure 2:**
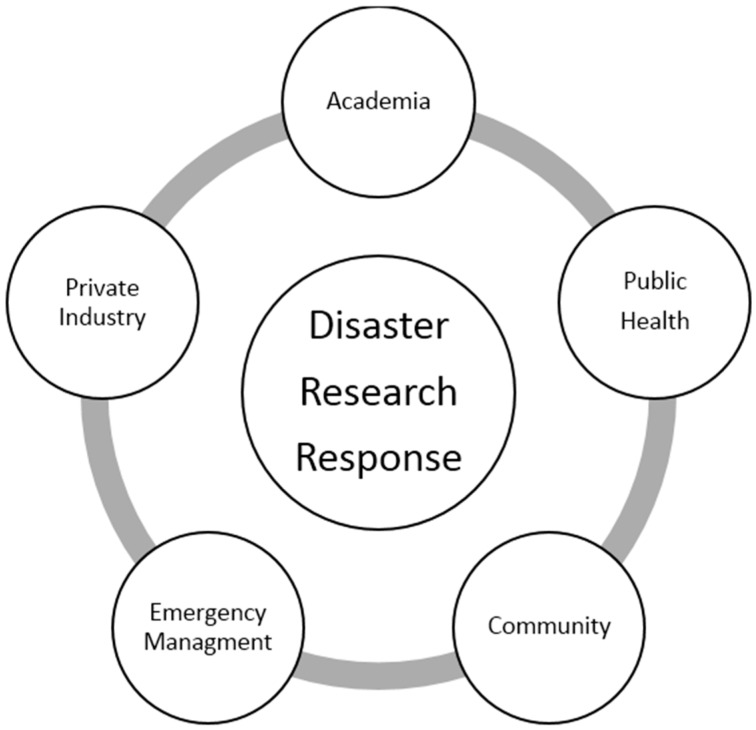
Disaster research partners.

The DR2 program promotes the concept of an integrated research response that seeks input from stakeholders as conceptualized in Figure 2.

**(6)**  **Integration of DR2 program efforts into existing federal and local planning, response, and recovery systems**

Integration with existing preparedness and response organizations is essential for the acceptance and success of disaster research. No function should have precedence over life-saving and worker safety activities. However, with the proper preparations, disaster research can be safely conducted early in the disaster response timeframe. Emergency managers who coordinate disaster response and recovery activities have a requirement to account for resources being used for every response-related activity. Likewise, public health officials need to know what health related activities are being performed during the response. Disaster researchers must understand the safety issues resulting from working in the disaster setting, as well as the established response structure. All groups share responsibility to prioritize and coordinate activities; manage resources; and to integrate information for decision-making and public communications. In the U.S., the use of the existing response and recovery infrastructure is currently the most effective way of coordinating efforts and sharing information across disciplines and jurisdictions and ensures that communications with community stakeholders are delivered in a timely manner. DR2 program staff have met with the various emergency management groups to inform them of the benefits of conducting research after disasters and educating them on how staff is being trained to operate in the disaster environment. DR2 program staff have also conducted briefings to federal agency partners on an annual basis, keeping them informed of DR2 program progress and has recently opened the discussion about developing a national framework for disaster research, much like the national frameworks for response and recovery [25,26]. These meetings have been an important venue for the coordination of efforts and the prevention of duplicative effort.

## 4. Future of DR2

There are many challenges to the successful implementation of the DR2 program. While a variety of elements are not directly controlled by NIEHS, such as the allocation of federal funds for disaster research, the Institute is actively engaged with various stakeholders to find solutions for these challenges. For example, NIEHS along with HHS and the Centers for Disease Control and Prevention (CDC) have co-funded a new National Academy of Sciences “Standing Committee on Medical and Public Health Research During Large-Scale Emergency Events” to provide a venue for discussion of issues related to short- and long-term strategic planning and how best to perform medical and public health disaster science research activities during a disaster. As needed, the standing committee will be involved in the planning, development, and oversight of related fast-track ad hoc committees to help prioritize scientific research needs during a public health event or in the immediate aftermath of a disaster [27].

The DR2 program continues to improve the state of environmental health disaster research. It is transitioning from a pilot project to a full-fledged program that will have increased cross-cutting impact across programs at NIEHS. Strategically, the DR2 program will continue to build out the EHN and empower academic institutions to build their own disaster research programs. Texas A & M University has announced the Texas One Gulf—a consortium of nine institutions that will be able to deploy disaster research response capability in the event of a Texas Gulf Coast disaster [28]. The DR2 Program will continue to encourage and assist other institutions to develop similar programs.

The NIH DR2 data collection tools repository and website will be refined to improve its functionality in an attempt to enable users to better access the information on the site. DR2 program staff will work with other organizations to expand the library of data collection tools and research protocols. Enhancing the capabilities and capacities of the EHN remains a priority. The vision is a nation-wide network of trained researchers who can serve as regional subject matter experts in environmental health supporting local and regional health departments and their surrounding communities. This will require effort with training, coordination, and communications with academic institutions and public health officials to better organize and integrate this resource into preparedness planning efforts. Of the highest priority and essential to the future success of the DR2 program is on-going coordination with academic researchers to share data and analyses with communities and vulnerable populations.

The DR2 program staff will also consider optimal ways to link environmental data to health effects by working with private industry and environmental protection agencies to obtain environmental contamination levels so those data can be linked to human exposure levels. Utilizing proprietary information from private industry to more accurately determine exposure levels and improve our understanding of applying and sharing that information are also central goals for the DR2 program.

The DR2 program staff continue to work on better integrating disaster research resources into response and recovery frameworks. Recent steps include meeting with emergency management officials to provide improved understanding of the advantages and benefits of disaster research. DR2 program staff are also looking at ways to train researchers so they can improve their understanding of the disaster setting and the frameworks by which disasters are managed. An ultimate goal is to create a national disaster research framework that builds upon the existing emergency management infrastructure, much like those for disaster response and recovery [25,26].

## 5. Conclusions

Improving our understanding of disasters requires a multidisciplinary approach of scientists from a diverse spectrum of social, behavioral, physical, and health sciences [29]. For disasters with an environmental health component, which are almost all disasters, systematic, integrated, interdisciplinary, and timely collection of data and analyses provides health officials with a prime opportunity to gain insights into the short- and long-term health consequences resulting from environmental exposures. In some cases, the health impacts are acute and disaster researchers can help identify significant health hazards and recommend effective interventions that are based on evidence collected in the immediate post-disaster period. However, many environmentally-related health impacts can only be determined through longitudinal studies and the concurrent capture and analysis of baseline data. Such data may only be accurate if obtained by researchers entering the field early on; collecting information from those exposed and pairing it with environmental exposure data. Pre-approved research protocols are a vital component due to the labor intensive and often complex nature of the efforts. Additionally, standing teams of trained researchers, and an outline of procedures or “Concept of Operations” that delineates roles and responsibilities for the operationalization of the research program would jump-start collection and storage of necessary data without impairing emergency response efforts.

Production of accurate, health relevant research demands integration into the established response and recovery mechanisms and organizational cultures. Gaining credibility and trust of the first responders is necessary for access to the disaster site and affected populations. For such relationships to occur, it is incumbent upon researchers to undergo training on the response system and how to work safely within a dangerous and austere disaster setting. Inclusion of community advocacy groups in the entire research process, from design to execution to research translation and communications, will assist in identifying health priorities and promoting trust and access to affected communities. Early steps to identify, contact, and discuss potential concerns and cultural sensitivities with vulnerable populations and community advocacy groups can provide enormous returns and facilitate the process when an event occurs.

The NIH DR2 program has addressed many of these issues in the context of environmental health. However, the DR2 program should not be viewed solely as a federal resource. Readily accessible data collection tools, such as those provided by the NIH DR2 website, enhance local capabilities to conduct disaster research without the need for federal support. NIEHS staff are available to provide technical assistance upon request. States have also begun to develop their own disaster research capacities as Texas has done in establishing the One Gulf program.

The goals of acute disaster surveillance and public health epidemiologic investigations are similar to longitudinal disaster research and closely intertwined. Both activities desire to turn health data into evidence-based public health and medical practice. In addition, both activities share many of the same challenges to data collection. In order to succeed all investigators need to work collaboratively, sharing their networks, experiences, findings, and collective wisdom to help answer the pressing questions of the day.

While the United States’ National Response and Recovery Frameworks do not specifically support research, these blueprints for disaster response and recovery do provide for wide-ranging government coordination and operational funding and can serve as important foundations for the development of a new “Framework for Strategic Research” in response to disasters. Thus, while efforts slowly evolve to develop such a Framework, disaster responders and researchers from all disciplines must collaborate to overcome the various and sometimes daunting challenges. Collection of timely scientific information will add to recovery efforts, enhance knowledge to improve future preparedness, and most importantly, help to reduce the adverse health impacts of disasters as they unfold.

## Figures and Tables

**Table 1:** DR2 collection tool repository: metadata descriptive fields.

Metadata Descriptive Fields	Metadata Descriptive Fields
Mode of Administration	Languages
Time to administer	Length/Number of questions
Population of interest	Author contact information
Copyright information	Access information
Interviewer training requirements	Purpose and Uses
History of use in a disaster setting	Reading Level
